# Reciprocal feedback inhibition of the androgen receptor and PI3K as a novel therapy for castrate-sensitive and -resistant prostate cancer

**DOI:** 10.18632/oncotarget.5659

**Published:** 2015-10-12

**Authors:** Wenqing Qi, Carla Morales, Laurence S. Cooke, Benny Johnson, Bradley Somer, Daruka Mahadevan

**Affiliations:** ^1^ West Cancer Center/University of Tennessee Health Science Center (UTHSC), Memphis, TN, USA

**Keywords:** hormone sensitive and resistant prostate cancer, androgen receptor, PI3K/AKT/mTOR, AR and PI3K/mTOR inhibitors resistant LNCaP cells

## Abstract

Gain-of-function of the androgen receptor (AR) and activation of PI3K/AKT/mTOR pathway have been demonstrated to correlate with progression to castration-resistant prostate cancer (CRPC). However, inhibition of AR or PI3K/mTOR alone results in a reciprocal feedback activation. Therefore, we hypothesized that dual inhibition of the AR and PI3K/mTOR pathway might lead to a synergistic inhibition of cell growth and overcome drug resistance in CRPC. Here, we reported that androgen-depletion increased AR protein level and Akt phosphorylation at Ser473 and Thr308 in LNCaP cells. Moreover, we developed resistance cell lines of LNCaP to Enzalutamide (or MDV3100), an AR inhibitor (named as LNCaP ‘MDV-R’) and PF-04691502, a PI3K/mTOR inhibitor (named as LNCaP ‘PF-R’). MTS analysis showed that LNCaP ‘PF-R’ was strongly resistant to Enzalutamide treatment, and on the other hand, LNCaP ‘MDV-R’ was 6-fold resistant to PF-04691502 treatment. Mechanistically, LNCaP ‘MDV-R’ cells had significantly reduced AR, loss of PSA and increase Akt activity in contrast with LNCaP ‘PF-R’ cells. Combined inhibition of PI3K/mTOR and AR pathways with a variety of small molecular inhibitors led to a synergistic suppression of cell proliferation and a profound increase of apoptosis and cell cycle arrest in both androgen-dependent LNCaP and independent CRPC 22Rv1 cell lines. In conclusion, this study provides preclinical proof-of-concept that the combination of a PI3K/mTOR inhibitor with an AR inhibitor results in a synergistic anti-tumor response in non-CRPC and CRPC models.

## INTRODUCTION

Prostate cancer is the number one incidence of cancer with 233,000 new cases and the second leading cause of cancer-related deaths in men in the United States [1]. Androgen-deprivation therapy is initially effective, but this therapy eventually fails and the disease progresses to castration-resistant prostate cancer (CRPC) [2–4]. Patients with CRPC have poor survival rates due to limited effective therapies. Despite recent phase III trials showing a survival advantage for abiraterone acetate (17α-hydroxylase/C17, 20-lyase inhibitor) and enzalutamide (AR inhibitor), the median duration of response is < 1 year [5, 6]. Several mechanisms including androgen receptor (AR) mutations, AR or its co-regulator proteins overexpression, intraprostatic androgen synthesis up-regulation and gene fusions have been proposed to result in CRPC progression, which suggests AR signaling remains a critical factor for the growth and survival of the hormone-refractory prostate cancer [7–15].

In addition to AR signaling, the PI3K/Akt/mTOR pathway is associated with prostate cancer. Uncontrolled activation of PI3K-signaling pathway through loss of PTEN has been found in about 40% of primary and 70% of metastatic prostate cancers [16–18]. Recently it was demonstrated that there is a bidirectional crosstalk between PI3K and AR survival pathways in PTEN-negative prostate cancers [19]. Inhibition of the PI3K pathway stimulates up-stream HER2/3 resulting in activation of the AR, whereas blockade of AR reduces FKBP5 levels impairing PHLPP function leading to upregulation of pAKT. Inhibition of the PI3K pathway alone causes growth arrest but not significant tumor regression in Pten-negative prostate cancers, however, combined PI3K and AR pathway inhibition gives profound tumor regressions. It has been reported that AZD5363, an AKT inhibitor potently inhibited proliferation and induced apoptosis in prostate cancer cell lines expressing the AR and had anticancer activity *in vivo* in androgen-sensitive and castration-resistant phases of a LNCaP xenograft model [20]. However, the effect of castration-resistant tumor growth inhibition and prostate-specific antigen (PSA) stabilization was transient and resistance occurred after approximately 30 days of treatment. Mechanistically, they found that single agent AZD5363 induced increase of AR binding to androgen response element, AR transcriptional activity, and AR-dependent genes such as PSA and NKX3.1 expression. These effects were overcome by the combination of AZD5363 with the antiandrogen bicalutamide, resulting in synergistic inhibition of cell proliferation and induction of apoptosis *in vitro*, and prolongation of tumor growth inhibition and PSA stabilization in CRPC *in vivo*.

In this study, we demonstrated that androgen-depletion increases AR expression and Akt activity. Further, we generated resistance cell lines of LNCaP to Enzalutamide (MDV3100) (LNCaP ‘MDV-R’), an AR inhibitor and PF-04691502 (LNCaP ‘PF-R’), a potent, selective pan-PI3K/mTOR inhibitor. MTS analysis showed that LNCaP ‘PF-R’ was strongly resistant to Enzalutamide however LNCaP ‘MDV-R’ was 6-fold resistant to PF-04691502. Mechanistically, we observed PI3K/Akt and AR pathway is activated in LNCaP ‘MDV-R’ and LNCaP ‘PF-R’, respectively. Combination targeting of PI3K/mTOR and AR pathways with a variety of small molecular inhibitors led to synergistic suppression of proliferation in both androgen-dependent LNCaP and independent 22Rv1 cell lines with increased apoptosis and cell cycle arrest. Together, this study provided a preclinical proof-of-concept that combination of a PI3K/mTOR inhibitor with an AR inhibitor resulted in an anti-tumor activity in both non-CRPC and CRPC models.

## RESULTS

### Androgen depletion increases AR protein level and Akt phosphorylation

**Figure 1:**
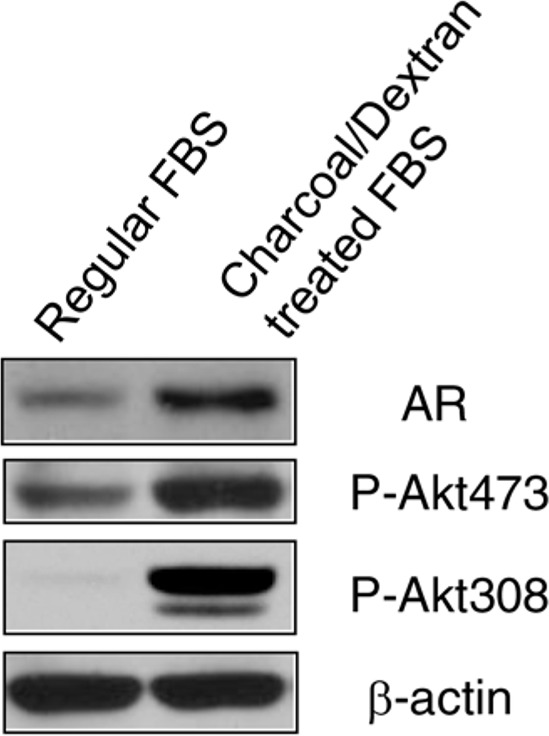
Depletion of androgen increased AR expression and Akt activity LNCaP cells were grown in regular RPMI 1640 medium with 10% FBS and androgen-depleted medium, phenol red-free RPMI 1640 supplemented with 10% charcoal/dextran-treated FBS for 10 days. Cells were lyzed and 50 μg total protein was resolved by electrophoresis on a 10% SDS-PAGE gel. Immunoblotting was performed using AR and phospho-Akt antibodies, respectively. β-actin protein was used as a loading control.

AR inhibitor resistance acquired in prostate cancer is associated with increased AR expression and PI3K/Akt activity. To further evaluate this observation, we grew LNCaP, an androgen-dependent cell line in androgen-depleted medium, phenol red-free RPMI 1640 supplemented with 10% charcoal/dextran-treated FBS for 10 days. Compared to LNCaP cells cultured in regular medium and serum, androgen depletion significantly resulted in increased AR protein and Akt phosphorylation at Ser473 and Thr308 (Fig. 1). These data suggest AR over-expression and Akt activity play important roles in resistance to androgen-deprivation therapy in prostate cancer.

### Acquired reciprocal resistance to PI3K/mTOR inhibition or AR inhibition in LNCaP cells

**Figure 2:**
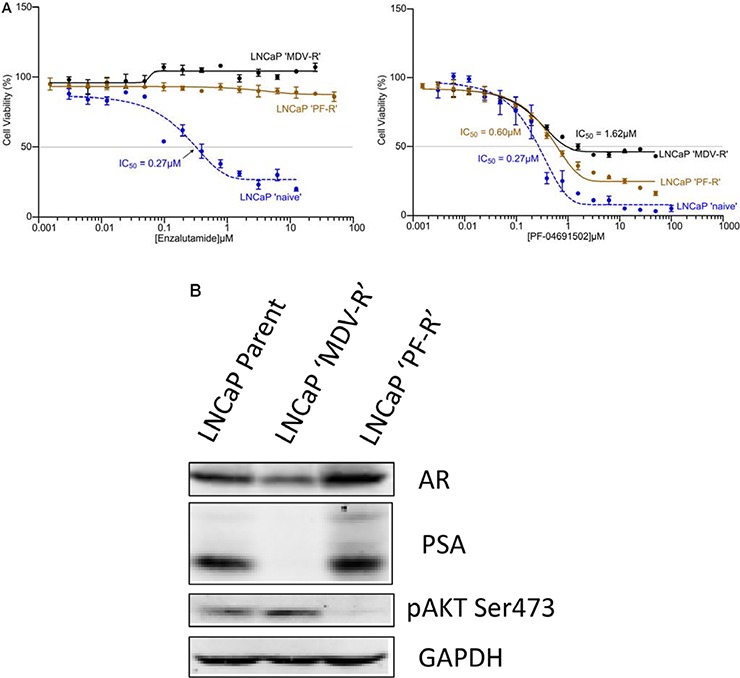
Acquired resistance to AR inhibitor and PI3K/mTOR inhibitor in LNCaP cells **A.** The AR or PI3K/mTOR inhibitor-resistant cell lines were developed by intermittent, incremental exposure of the LNCaP cells to various concentrations (5–10 μM) of Enzalutamide or 0.1–0.3 μM of PF-04691502 for 12 months designated as LNCaP ‘MDV-R’ and LNCaP ‘PF-R’ respectively. The LNCaP ‘MDV-R’, LNCaP ‘PF-R’ and parental LNCaP cell lines were evaluated in an MTS assay for cell viability at Enzalutamide and PF-04691502 concentrations varying from 0.001 to 100 μM. **B.** The parent LNCaP, LNCaP ‘MDV-R’ and LNCaP ‘PF-R’ cells were lysed and immunoblotting was performed to detect AR, PSA and phosphorylation of Akt. GAPDH was used as a loading control.

To further examine the role of AR and PI3K/Akt/mTOR signaling, we generated both AR and PI3K/mTOR inhibitor resistant LNCaP cell lines. Parental LNCaP cells were exposed intermittently and incrementally to various concentrations Enzalutamide (MDV3100) (5–10 μM) or PI3K/mTOR inhibitor PF-04691502 (0.1–0.3 μM) for 12 months. The LNCaP cells finally became stable and can grow in 10 μM of Enzalutamide or 0.3 μM of PF-04691502 which were designated LNCaP ‘MDV-R’ or LNCaP ‘PF-R’, respectively. The LNCaP ‘MDV-R’, LNCaP ‘PF-R’ and parental LNCaP cell lines were evaluated by MTS assays for cell viability at Enzalutamide and PF-04691502 concentrations varying from 0.001 to 100 μM. As expected, LNCaP ‘MDV-R’ cells were completely resistant to Enzalutamide and 25% LNCaP ‘PF-R’ cells were resistant to PF-04691502 (Fig. 2A). Interestingly, LNCaP ‘PF-R’ cells were totally resistant to Enzalutamide and ~50% LNCaP ‘MDV-R’ cells were resistant to PF-04691502 (Fig. 2A). Consistent with this observation, loss of AR expression, loss of PSA and activation of Akt signaling were identified in LNCaP ‘MDV-R’ but the opposite effect was seen in LNCaP ‘PF-R’ (Fig. 2B). These results demonstrate the existence of a reciprocal feedback activation pathway between the AR and the PI3K/mTOR pathway in prostate cancer.

### Suppression of PI3K/mTOR inhibits cell proliferation in AR inhibitor sensitive and resistant prostate cancer cell lines

**Figure 3:**
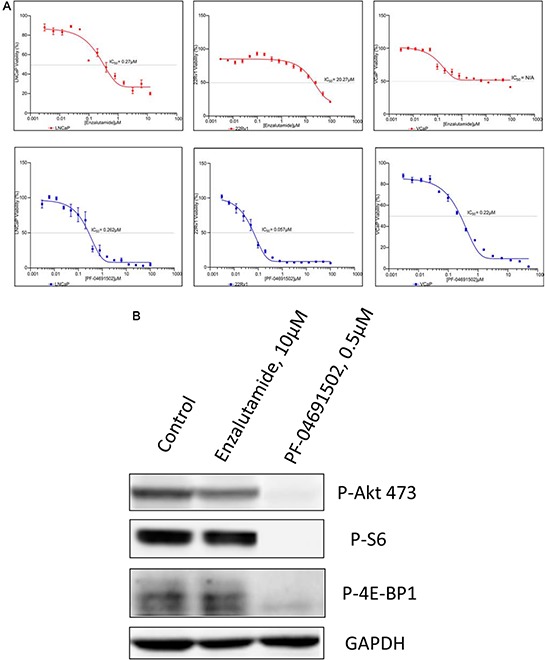
Antiproliferative activity of PI3K/mTOR inbibitor and AR inhibitor in prostate cancer cell lines **A.** LNCaP, 22Rv1 and VCaP cells were exposed to varying concentrations of Enzalutamide or PF-04691502 for 4 days. Cell viability was assessed by MTS analysis. Points are the means of triplicate determinations ± SD. Inset: The IC_50_s of Enzalutamide and PF-04691502 were calculated in these cell lines and shown. **B.** LNCaP cells were untreated or treated with 10 μM of Enzalutamide and 0.5 μM of PF-04691502 for 18 h. Cells were collected for protein isolation. 50 μg of total protein from each lysate was resolved by SDS-PAGE and immunoblotted with antibodies specific for phosphorylated Akt (Ser473), phosphorylated S6 and phosphorylated 4E-BP1. GAPDH was used as a loading control.

The PI3K pathway plays a central role in cancer cell survival and proliferation across a variety of malignancies and has been validated as a drug target for cancer therapy [21, 22]. To examine whether suppression of PI3K pathway inhibits prostate cancer cells, MTS assays were performed to evaluate the growth in LNCaP, 22Rv1 and VCaP cell lines. First, we evaluated two drugs, Enzalutamide and Abiraterone and found that the IC_50_ of Enzalutamide and Abiraterone were 0.27 μM and 10.49 μM in LNCaP cells, 20.27 μM and 25.47 μM in 22Rv1 cells, and unreached IC_50_ and 30.32 μM in VCaP cells, indicating LNCaP cells were sensitive to AR inhibition but 22Rv1 and VCaP were not (Fig. 3A and Table 1). However, except LY294002, the other three PI3K/mTOR inhibitors GDC-0980, GDC-0941 and PF-04691502 effectively inhibited the growth of these three cell lines with IC_50_ values of 0.06 μM to 0.43 μM (Fig. 3A and Table 1). Moreover, to determine whether these inhibitors specifically inhibited the PI3K pathway, we treated LNCaP cells with a representative drug PF-04691502. Immunoblotting showed complete suppression downstream of PI3K/mTOR, including Akt, S6 and 4E-BP1 (Fig. 3B). Together, the data demonstrate that blockage of PI3K/mTOR pathway successfully suppress cell proliferation in both AR sensitive and non-sensitive prostate cancer cell lines.

**Table 1:** IC_50s_ (μM) of PI3K/mTOR and AR signaling inhibitors in castration-sensitive LNCaP and castration-resistant 22Rv1 and VCaP cell lines

Inhibitors Cell lines	GDC-0980	GDC-0941	LY294002	PF-04691502	Enzalutamide	Abiraterone
LNCaP	0.32	0.40	23.63	0.26	0.27	10.49
22Rv1	0.13	0.43	16.91	0.06	20.27	25.47
VCaP	0.21	0.39	19.30	0.22	N/A	30.32

### Dual inhibition of PI3K/mTOR and AR pathways led to synergistic suppression of proliferation and increased apoptosis in LNCaP and castration-resistant 22Rv1 and VCaP cell lines

**Figure 4:**
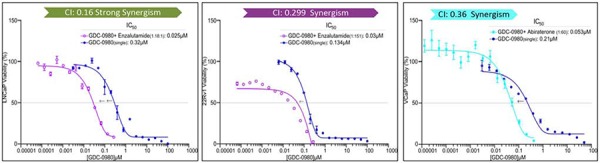
PI3K/mTOR inhibitor synergizes with AR inhibitor to decrease cell viability LNCaP, 22Rv1 and VCaP cells were treated with PI3K/mTOR inhibitor, GDC-0980 plus AR signaling inhibitor, Enzalutamide for 4 days. The dose ratio was calculated for each combination based on the IC_50_ of single agents. MTS assays were carried out and the combination-index (CI) was calculated. Representative graphs were shown for the synergistic effect of each combination treatment.

**Figure 5:**
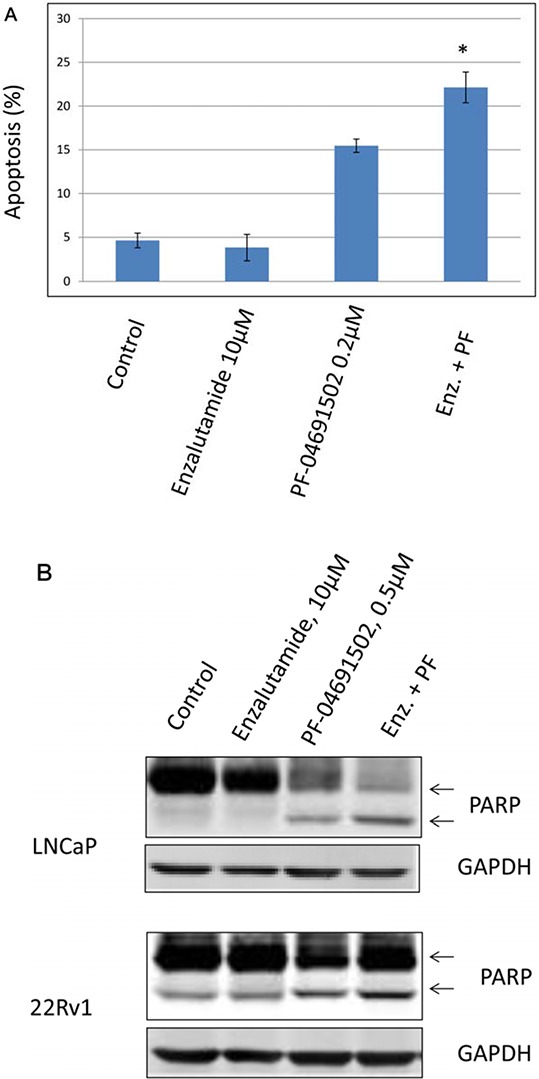
The combination treatment of Enzalutamide and PF-04691502 increases apoptosis in LNCaP and 22Rv1 cells **A.** LNCaP cells were treated with 10 μM of Enzalutamide (Enz.) or 0.2 μM of PF-04691502 (PF) or 10 μM of Enzalutamide combined with 0.2 μM of PF-04691502 for 72 h. Apoptosis was analyzed by flow cytometry after annexin V and PI staining. Columns show means of triplicate analysis ± S.D. *, *P* < 0.05 differ from control and single agents by Student *t* test. **B.** LNCaP and 22Rv1 cells were treated with 10 μM of Enzalutamide or 0.5 μM of PF-04691502 or 10 μM of Enzalutamide combined with 0.5 μM of PF-04691502 for 72 h. PARP-cleavage levels were analyzed by Western blotting. GAPDH was used as a loading control.

Since inhibition of the AR pathway results in activation of the PI3K/Akt/mTOR pathway by reciprocal feedback activation, we hypothesized that dual inhibition of PI3K/mTOR and AR has synergistic inhibition of cell proliferation and may overcome drug resistance. As expected, a variety of combinations of PI3K/mTOR and AR inhibitors resulted in synergism or strong synergism of inhibition of cell viability in not only LNCaP, but also in CRPC cell lines 22Rv1 and VCaP (Fig. 4 and Table 2). It is known that apoptosis is induced when the PI3K pathway is suppressed. To examine apoptosis, LNCaP cells were treated with 10 μM of Enzalutamide or 0.2 μM of PF-04691502 or 10 μM of Enzalutamide combined with 0.2 μM of PF-04691502 for 72 hours, stained with Annexin V and PI and evaluated by flow cytometry. PF-04691502 but not Enzalutamide induced apoptosis in LNCaP cells (Fig. 5A). However, apoptosis was more prominent with combination treatment (Fig. 5A). These results were confirmed by demonstrating an increased level of cleaved PARP in both LNCaP and 22Rv1 cells (Fig. 5B). Enzalutamide did not induce PARP cleavage, however, PF-04691502 alone led to PARP cleavage and combination of Enzalutamide and PF-04691502 resulted in additional PARP cleavage. In summary, dual targeting of PI3K/mTOR and AR resulted in synergistic inhibition of cell proliferation and significant induction of apoptosis in castration-sensitive LNCaP and castration-resistant 22Rv1 and VCaP cell lines.

**Table 2:** Combination indices derived from the median-effect principle of Chou and Talalay for different combination treatments in prostate cancer cell lines

Combinations Cell lines	GDC-0980+ Enzalutamide	GDC-0941+ Enzalutamide	LY294002+ Enzalutamide	PF-04691502+ Enzalutamide	GDC-0980+ Abiraterone	GDC-0941+ Abiraterone	LY294002+ Abiraterone	PF-04691502+ Abiraterone
LNCaP	0.16	0.36	0.31	0.50	0.30	0.30	0.30	0.04
22Rv1	0.30	0.14	0.24	0.46	0.26	0.21	0.39	0.42
VCaP	N/A	N/A	N/A	N/A	0.36	0.36	0.32	0.21

### AR inhibitor and PI3K/mTOR inhibitor induced G1 and G2 cell cycle arrest in prostate cancer cells

**Figure 6:**
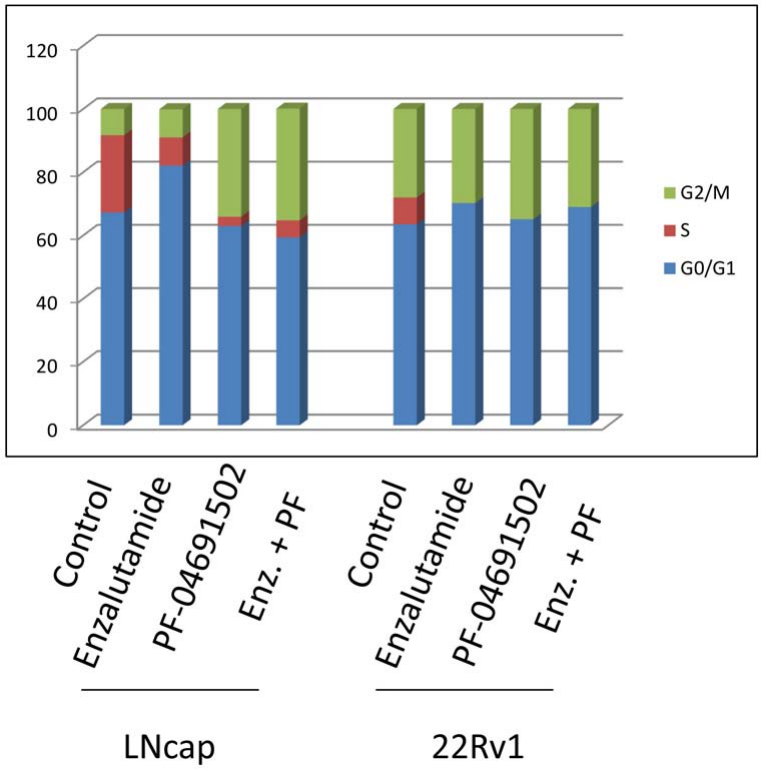
AR and PI3K/mTOR inhibitor induced cell cycle arrest in prostate cancer cells LNCaP and 22Rv1 cells were treated with 10 μM of Enzalutamide or 0.2 μM of PF-04691502 or combination of 10 μM of Enzalutamide and 0.2 μM of PF-04691502 for 48 h. Samples were harvested and stained with propidium iodide. DNA contents were evaluated by flow cytometry. Percentages of G0/G1, S and G2/M phase cell populations were analyzed by ModFit software and graphed.

Activation of the AR or PI3K pathway regulates cell cycle progression. To examine cell cycle progression affected by the AR or PI3K/mTOR inhibitor in prostate cancer cells, LNCaP and 22Rv1 cells were treated with 10 μM of Enzalutamide or 0.2 μM of PF-04691502 or 10 μM of Enzalutamide combined with 0.2 μM of PF-04691502 for 48 hours and DNA content was evaluated using flow cytometry (Fig. 6). Treatment with Enzalutamide dramatically increased G0/G1 and decreased S phase populations in both cell lines. However, treatment with PF-04691502 induced G2/M arrest and also reduced S populations. Similarly, combination treatment induced G2/M arrest in LNCaP cells and continued to reduce S phase in both of cell lines. These effects may contribute to inhibition of cell proliferation and induction of apoptosis by AR and PI3K/mTOR inhibitors in these cells.

## DISCUSSION

Patients with castrate-resistant prostate cancer (CRPC) have poor survival rates due to limited effective therapies. Gain-of-function of the androgen receptor (AR) and activation of PI3K/Akt/mTOR signaling pathway correlate with progression to castration-resistant prostate cancer (CRPC). However, as single agents, AR or PI3K/mTOR inhibitors result in a reciprocal feedback activation loop. Therefore, combination of a PI3K/mTOR inhibitor with an AR inhibitor might result in a more profound suppression of CRPC. In this study, we demonstrated that acute depletion of androgen increased AR expression and Akt activity in LNCaP cells. Further, chronic inhibition of AR by Enzalutamide (MDV3100) resulted in a decrease of AR protein level and loss of AR activity implicated by a markedly reduced PSA expression, increased Akt phosphorylation, and resistance to AR and PI3K/mTOR inhibitors. On the other hand, chronic inhibition of PI3K/mTOR pathway by PF-04691502, a potent, selective pan-PI3K/mTOR inhibitor resulted in suppression of Akt activation, increased AR and PSA expression, and robust resistance to Enzalutamide. Inhibition of PI3K/mTOR but not AR alone suppressed cell proliferation in CRPC cell lines 22Rv1 and VCaP. However, combination of PI3K/mTOR and AR led to synergistic suppression of proliferation and induction of further apoptosis and cell cycle arrest.

It has been reported that inhibition of the PI3K pathway increased AR protein levels and its target gene activity in PTEN-negative prostate cancers. Conversely, blockade of AR resulted in activation of PI3K. Combined PI3K and AR pathway inhibition gave profound tumor regressions in preclinical models of prostate cancer [19]. It is also reported that the AKT inhibitor AZD5363 potently inhibits proliferation and induces apoptosis in prostate cancer cell lines and has anticancer activity *in vivo* in androgen-sensitive and castration-resistant phases of the LNCaP xenograft model. However, the effect of castration-resistant tumor growth inhibition and prostate-specific antigen (PSA) stabilization is transient and resistance occurs with increasing PSA after approximately 30 days of treatment. The combination of AZD5363 with the antiandrogen bicalutamide results in synergistic inhibition of cell proliferation and induction of apoptosis *in vitro*, and prolongation of tumor growth inhibition and PSA stabilization in CRPC *in vivo* [20]. Consistent with these reports, our results support the combined inhibition of PI3K/mTOR and AR to represent a novel therapeutic strategy that warrants clinical trial evaluation in patients with CRPC. Currently there are two clinical trials investigating PI3Kβ inhibitor GSK2636771 (NCT02215096) and pan-PI3K/mTOR inhibitor LY3023414 (NCT02407054) in combination with enzalutamide in metastatic castration-resistant prostate cancer patients.

The PI3K/Akt/mTOR pathway is frequently activated and plays a central role in tumorigenesis across a variety of malignancies [21, 22]. It is upregulated and has been implicated in the survival and metastasis of prostate cancer cells, especially in high Gleason score and in CRPC [23, 24]. Thus, selective targeting of this pathway may provide opportunities to affect prostate cancer growth. Currently, several small-molecule inhibitors targeting different proteins of the PI3K/AKT/mTOR pathway, especially a class of dual PI3K/mTOR inhibitors, which bind to and inactivate both PI3K and mTOR, have shown potent anticancer activity in prostate cancer in pre-clinical and clinic trials [25, 26] (Table 3). PF-04691502 and GDC-0980 are novel, potent, selective class I PI3K and mTOR inhibitors [27, 28]. GDC-0941 is a potent inhibitor of PI3Kα/δ [29]. All three drugs have entered phase I or II clinical trials [30, 31]. Our data here indicated that GDC-0980, GDC-0941 or PF-04691502 alone efficiently inhibited cell proliferation (Fig. 3A) with inactivation of the PI3K pathway, including Akt, S6 and 4E-BP1 (Fig. 3B), and induced apoptosis in both castration-sensitive LNCaP cells and castration-resistant 22Rv1 and VCaP cells. In contrast, AR-resistant LNCaP cells (LNCaP ‘MDV-R’) are insensitive to PI3K/mTOR inhibition (Fig. 2A). Similar to the AKT inhibitor, anti-tumor effects are likely transient and resistance occurs after chronic exposure. However, combinations of LY294002, GDC-0980, GDC-0941 or PF-04691502 with AR signaling inhibitor, Enzalutamide or Cyp450 17A1 inhibitor Abiraterone significantly delays CRPC growth associated with induction of enhanced apoptosis compared to single agent therapy. Hence, a combination is likely to be more effective than monotherapy and also may reduce drug dosage to minimize side effect to normal tissue in elderly patients with CRPC.

**Table 3:** Summary of drugs targeting the PI3K and AR pathways

Inhibitor	Target	Reference
GDC-0980	**PI3Kα/β/δ/γ**	28
GDC-0941	**PI3Kα/δ**	22; 29; 30
LY294002	**PI3Kα/δ/β**	26
PF-04691502	**PI3K(α/β/δ/γ)/mTOR**	27; 31
Enzalutamide	**Androgen-Receptor (AR)**	6
Abiraterone	**Cyt P450-17A1**	5

Studies of cell cycle progression in prostate cancer cells have shown that androgen is a critical regulator of the G1-S transition. Mechanistic investigation has revealed that AR mediates cdk4/6 activation and subsequent phosphorylation and inactivation of the retinoblastoma tumor suppressor (RB) to promote G1-S phase progression and thereby govern androgen-dependent proliferation [32, 33]. Knudsen *et al*. and Xu *et al*. reported that prostate cancer cells deprived of androgen arrest in early G1 phase [34, 35]. Similarly, our data in this study show inhibition of AR by Enzalutamide dramatically blocked prostate cancer cells entering S phase from G1 (Fig. 6), especially in androgen-dependent LNCaP cells. Interestingly, the PI3K/Akt/mTOR signaling has been implicated to regulate both G1/S and G2/M transition [36]. Inhibition of PI3K by LY294002 or other PI3K inhibitors and mTOR by rapamycin have been demonstrated to induce G1 cell cycle arrest in the prostate cancer cells [37] and other human malignances [38, 39]. However, recently LY294002 has been shown to block G2/M transition in retinal cells [40]. The novel PI3K/mTOR inhibitors, GDC-0980, GDC-0941 and PF-04691502 have been reported to induce G1 cell-cycle arrest in breast, lung, glioblastoma and hepatocellular carcinoma cells [27, 41–43]. In contrast, here exposure to PF-04691502 markedly resulted in G2/M arrest in both castration-sensitive and resistant cell lines (Fig. 6). The detailed molecular mechanisms need further investigation.

In conclusion, our findings indicate that disruption of AR signaling by androgen depletion or AR inhibitor upregulate the PI3K/Akt/mTOR pathway and subsequently acquire resistance to PI3K/mTOR inhibitor. Conversely, chronic inhibition of the PI3K/Akt/mTOR pathway acquires resistance to AR inhibitor therapy. Suppression of PI3K/mTOR alone results in inactivation of Akt, S6 and 4EBP1, reduction of cell proliferation induction of apoptosis and G2/M cell cycle arrest. Most important, we show that dual inhibition of AR and PI3K/mTOR signaling pathways has synergistic inhibitory effect of cell growth associated with increase of apoptosis in both castration-sensitive and resistant prostate cancer cells. These results suggest that PI3K/mTOR axis should be inhibited in combination with AR inhibition in hormone sensitive and CRPC in early therapeutic trials.

## MATERIALS AND METHODS

### Cells and reagents

LNCaP, Vcap and 22Rv1 cell lines used in this study were from ATCC (Rockville, MD) and maintained in RPMI 1640 medium (Mediatech, VA) supplemented with 10% fetal bovine serum, 2 mM sodium pyruvate and 100 units/ml penicillin/streptomycin at 37°C in a humidified atmosphere containing 5% CO2. For the androgen-depletion experiments, LNCaP cells were grown in androgen-depleted medium, phenol red-free RPMI 1640 supplemented with 10% charcoal/dextran-treated FBS (HyClone, Logan, UT). For resistance cell lines development, the LNCaP cells were exposed intermittently and incrementally to various concentrations (5–10 μM) of Enzalutamide or 0.1–0.3 μM of PF-04691502. A total of 12 months later, LNCaP cells were grown stably in Enzalutamide (10 μM) or PF-04691502 (0.3 μM)-containing medium, and these resistant cells were evaluated in an MTS assay for cell viability and labeled LNCaP ‘MDV-R’ and LNCaP ‘PF-R’. AR inhibitor Enzalutamide was provided by Medivation, Inc. and Astellas Pharma Inc. Abiraterone and PI3K/mTOR inhibitors: GDC-0980, GDC-0941, LY294002 and PF-04691502 were purchased from http://Selleckchem.com (Houston, TX). The compounds were dissolved at 50 mM in DMSO as a stock solution, and then further diluted to desired concentrations for *in vitro* experiments. Anti-AR, Anti-PSA and anti-PARP (H-250) antibodies were purchased from Santa Cruz Biotechnology (Santa Cruz, CA). Anti-phospho-Akt (Ser473 and Thr308), anti-phospho-S6, anti-phospho-4E-BP1 and anti-GAPDH (14C10) antibodies were from Cell Signaling Technology (Danvers, MA), and anti-β-actin antibody was purchased from Sigma (St Louis, MO).

### Cell proliferation assay (MTS assay)

Cells were seeded at 10,000 per well in 96-well culture plates and allowed to grow for 24 hr followed by the desired treatment with increasing concentrations of the indicated agents (Enzalutamide, Abiraterone, GDC-0980, GDC-0941, LY294002 and PF-04691502) for 4 days. Viable cell densities were determined using a CellTiter 96 Cell Proliferation Assay kit (Promega). Absorbance readings at 490 nm were analyzed against the control group for each drug treatment to determine cell viability. The studies were performed in triplicates x 4 and IC_50_ values were estimated by Calcusyn software (Biosoft, UK). For combination studies of AR inhibition plus PI3K/mTOR inhibition, an equipotent ratio was calculated to determine a combined graded combination treatment. The equipotent ratio is the ratio of the median effects resulting from the single dose treatments of AR inhibitors and PI3K/mTOR inhibitors. A control group was established for each drug treatment in six replicates. The effects of the combined treatments were determined by the combination-index (CI) and isobologram methods derived from the median-effect principle of Chou and Talalay.

### Apoptosis assay

Cells were treated with 10 μM of Enzalutamide or 0.2 μM of PF-04691502 or combination of 10 μM of Enzalutamide and 0.2 μM of PF-04691502 for 72 h. Using Annexin V staining to detect apoptosis, treated cells were harvested and rinsed with cold PBS once. After centrifugation for 5 min, cells were resuspended in 500 μl of 1x Annexin V binding buffer (BioVision, Annexin V-FITC Reagent Kit, Cat.#1001–1000) and then added 5 μl of Annexin V-FITC and 5 μl of Propidium Iodide (BioVision, Annexin V-FITC Reagent Kit). After incubation for 5 min at room temperature in the dark, the samples were analyzed by flow cytometry.

### Cell Cycle analysis

LNCaP and 22Rv1 cells were treated with 10 μM of Enzalutamide or 0.2 μM of PF-04691502 or combination of 10 μM of Enzalutamide and 0.2 μM of PF-04691502 for 48 h. Treated cells were centrifuged at 1,500 g for 5 min at 4°C and resuspended in PBS, fixed by drop-wise addition of ice-cold ethanol (100%) to a final concentration of 70%, and incubated for 30 min on ice. Fixed cells were pelleted and treated with 100 μl of RNase A (0.2 mg/ml in PBS) for 5 min at room temperature, then suspended in 0.5 ml ddH2O. After staining with 4 μg/ml propidium iodide, the DNA content was determined using a Becton Dickson flow cytometer and the cell cycle profile was analyzed by ModFit software. Cell aggregates were gated out of the analysis, based on the width of the propidium iodide fluorescence signal. Each profile was compiled from 10,000 gated events.

### Immunoblotting

The cells were lysed in NP-40 lysis buffer containing 50 mM Tris.Cl (pH 7.4), 0.15 M NaCl, 0.5% NP-40, 1 mM DTT, 50 mM Sodium Fluoride, and 2 ml/ml Protease inhibitor cocktail (Sigma, St. Louis, MO). Protein concentrations were determined using the BioRad protein assay kit (Hercules, CA) and 50 μg of protein was resolved by electrophoresis on a 10% SDS-PAGE gel. The proteins were then transferred onto a nitrocellulose membrane and non-specific binding was blocked by incubating with 5% nonfat milk in TBST buffer (0.01 M Tris-Cl, 0.15 M NaCl, 0.5% Tween-20, pH 8.0) at room temperature for 1 hr. The membrane was subjected to the indicated antibodies and the proteins were detected by a LI-COR Odyssey Infrared Imaging System.

### Statistical analysis

All *in vitro* experiments were performed in triplicate. The results were expressed as mean± S.D. The difference between two mean values were measured by the Student's *t*-test (Excel) and considered to be statistically significant when *p* ≤ 0.05.
